# Study design and protocol for evaluating the long‐term prognosis of patients receiving his bundle pacing: A multicenter observational study

**DOI:** 10.1002/joa3.12229

**Published:** 2019-08-16

**Authors:** Satoshi Yanagisawa, Yasuya Inden, Hiroyuki Kato, Hirohiko Suzuki, Masaya Fujita, Shinji Ishikawa, Yasunori Kanzaki, Yosuke Kamikubo, Yosuke Murase, Toyoaki Murohara, Rei Shibata

**Affiliations:** ^1^ Department of Advanced Cardiovascular Therapeutics Nagoya University Graduate School of Medicine Nagoya Japan; ^2^ Department of Cardiology Nagoya University Graduate School of Medicine Nagoya Japan; ^3^ Division of Cardiology Japan Community Healthcare Organization Chukyo Hospital Nagoya Japan; ^4^ Department of Cardiology, Cardiovascular Center Nagoya Daini Red Cross Hospital Nagoya Japan; ^5^ Department of Cardiology Toyota Kosei Hospital Toyota Japan; ^6^ Department of Cardiology, Cardiovascular Center Anjo Kosei Hospital Anjo Japan; ^7^ Department of Cardiology Ogaki Municipal Hospital Ogaki Japan; ^8^ Division of Cardiology Toyota Memorial Hospital Toyota Japan; ^9^ Department of Cardiology Komaki City Hospital Komaki Japan

**Keywords:** atrioventricular block, His bundle pacing, pacemaker, prognosis

## Abstract

**Background:**

His bundle pacing (HBP) is a recently developed pacing technique that can achieve an ideal physiological pattern of ventricular activation via stimulation of the native His‐Purkinje system. Despite the widespread introduction of HBP in clinical practice, its appropriate indications are yet to be determined clearly. Moreover, the efficacy and safety of HBP and long‐term prognosis of patients undergoing such are unknown.

**Methods:**

We conducted a multicenter observational prospective study in patients undergoing HBP in Japan. Patients with atrioventricular block or conduction delay and estimated ventricular pacing of ≥ 40% scheduled for HBP implantation are included. All patients are followed up until 3 years after the implantation. The primary endpoints are all‐cause death, heart failure‐related hospitalization, and upgrade to cardiac resynchronization therapy. The secondary endpoint is changes in cardiac function based on echocardiographic findings and laboratory data after the implantation.

**Results:**

The results are currently under investigation.

**Conclusions:**

This multicenter observational study evaluates the long‐term prognosis and changes in cardiac function of patients undergoing HBP implantation in a clinical setting. Considering the large number of patients included, the cumulative results would be helpful in establishing evidence on HBP application in this area and consequently allow accurate management and treatment of patients undergoing HBP.

## 1 INTRODUCTION

His bundle pacing (HBP) is a recently developed pacing technique used in clinical practice. It aims to achieve an ideal physiological pattern of ventricular activation by stimulating the native His‐Purkinje system via fixation of the lead directly to the conduction system. Compared with conventional right ventricular pacing (RVP), HBP can shorten the pacing QRS duration and induce physiological ventricular activation, resulting in reduction of ventricular and atrioventricular dyssynchronies and hemodynamic improvement.^1^ The clinical application of HBP in humans has been recently initiated; the use of recently introduced and specially designed pacing lead and sheath can help in performing HBP with reasonable success rates.^2^, ^3^

To date, several outcomes supporting the use of HBP have been gradually reported; however, only a few studies have investigated this technique. HBP has been reported to improve the New York Heart Association classification, 6‐minute walk test findings, quality of life, and left ventricular ejection fraction (LVEF) when compared to that achieved with RVP in crossover‐design studies.^4^, ^5^ A recent study demonstrated that HBP was associated with a significant decrease in hospitalizations owing to heart failure, especially in patients requiring ventricular pacing at >20%, in comparison with RVP.^6^ The updated ACC/AHA/HRS practice guideline suggested that using HBP and cardiac resynchronization therapy (CRT) is reasonable in patients with atrioventricular block as an indication for permanent pacing with an LVEF of 36%‐50% and expected ventricular pacing of >40%.^7^ Additionally, HBP may serve as an alternative therapeutic option in patients with bundle branch block and heart failure with reduced ejection fraction, which is a traditional indication for CRT.^8^, ^9^, ^10^, ^11^ Left bundle branch block was successfully corrected with QRS duration narrowing with the use of HBP in a large number of patients recent study.^8^ This may be based on the hypothesis that the His bundle may have a longitudinal dissociation separated by the right and left bundle branches at the main His trunk.^12^ His conduction system injury may be located in relatively paroxysmal sites, and stimulation of the distal site using pacing lead over the injury site could correct conduction delays, improving the QRS duration.

However, the abovementioned results were reported in very recent studies with a nonrandomized, a crossover, or an observational design. These studies were conducted in hospitals with well‐experienced trained experts who have treated a large number of patients with HBP.^13^, ^14^, ^15^, ^16^ It is unknown whether the high success rate of HBP implantation (≥80%‐90%) in hospitals would be similar to that in other nontertiary hospitals in clinical settings. Moreover, the follow‐up period after HBP was relatively short, and the long‐term outcomes of HBP remain to be elucidated. In addition, this procedure has some disadvantages. An increased pacing threshold in HBP was more likely to be observed after the follow‐up period, which could cause early battery loss, early generator replacement, and lead revisions.^13^ In progressive conduction diseases, including cardiac sarcoidosis, amyloidosis, and acute myocardial infarction, future occurrence of distal site blocks from the pacing site after implantation may be expected, raising concerns regarding the permanent stability of HBP.

Despite the widespread introduction of HBP in clinical practice, its appropriate indications are yet to be determined clearly. Therefore, a multicenter prospective study on HBP in a large sample with a long follow‐up period in the real‐world clinical setting was needed. Thus, this study was prospectively conducted to evaluate the prognosis of patients receiving HBP in multiple centers. We also assess the changes in cardiac function after HBP implantation in these patients.

## 2 METHODS

### 2.1 Objective

This study was designed as a multicenter prospective observational study (UMIN Clinical Trials Registry; UMIN 000,035,534). The objective of the study was to evaluate the long‐term prognosis and changes in cardiac function of patients undergoing HBP implantation in a clinical setting.

### 2.2 Study population

This multicenter study was conducted in eight institutions in Japan (Nagoya University Hospital, Chukyo Hospital, Nagoya Daini Red Cross Hospital, Toyota Kosei Hospital, Anjo Kosei Hospital, Ogaki Municipal Hospital, Toyota Memorial Hospital, and Komaki City Hospital). These institutions have been certified as institutions of the Japanese Heart Rhythm Society. In all institutions, device implantation is routinely performed by an electrophysiology specialist. At least one physician in each hospital has received a standard lecture for the management and implantation of HBP before use. Nagoya University Hospital was the representative institution among the participating institutions in this study.

The inclusion criteria were as follows: (a) eligibility to receive permanent pacemaker therapy for atrioventricular block or conduction delay, including complete bundle branch block, and scheduled for HBP implantation, (b) estimated ventricular pacing of ≥40% after the follow‐up period, and (c) consent to participate in this study. The indications used for pacemaker implantation for atrioventricular block and conduction delay were in compliance with the recent guidelines.^7^, ^17^

From April 2019, patient inclusion was started prospectively after approval of the ethical committee of each hospital. All patients provided written or opt‐out informed consent for inclusion in the study and HBP implantation. Patient inclusion will be performed up to 3 years (until March 2022). This study was performed in compliance with the Declaration of Helsinki principles. The study protocol was approved by the institutional ethics committees of all hospitals.

### 2.3 Baseline evaluation

The baseline demographic and characteristics of the patients are assessed. Echocardiographic findings (ie, LVEF, left atrial diameter, left ventricular endo‐diastolic/‐systolic diameter, and left ventricular endo‐diastolic/‐systolic volume) and laboratory data (ie, B‐type natriuretic peptide level or NT‐pro B‐type natriuretic peptide level) are also assessed. The detailed parameters are shown in Table 1. During the HBP procedure, HBP parameters, including His bundle threshold, R‐wave sensing, QRS duration, pacing morphology (selective HBP/nonselective HBP/RVP), and procedure time are evaluated (Table 1). The patients’ data are input in an electronic data capture system that created by Mebix, Inc., Tokyo, Japan. All data are collected and managed with anonymization for principal investigators and coinvestigators in all institutions.

**Table 1:** Demographic and baseline characteristics and parameters at implantation

Characteristics/parameters
Age, year
Male sex
Height/weight, cm/kg
Etiology and diagnosis
Coronary heart disease (angina pectoris and myocardial infarction)
Congestive heart failure
Atrial fibrillation (paroxysmal, persistent, and chronic)
Sarcoidosis
Cardiomyopathy (dilated cardiomyopathy and hypertrophic cardiomyopathy)
None
Echocardiographic finding
Left ventricular ejection fraction, %
Left atrial diameter, mm
Left ventricular endo‐diastolic/‐systolic diameter, mm
Left ventricular endo‐diastolic/‐systolic volume, mL
Laboratory data
B‐type natriuretic peptide level, pg/dL
NT‐pro B‐type natriuretic peptide level, pg/dL
Type of block
Atrioventricular block (atrioventricular nodal block, intra His bundle block, and infra His bundle block)
Complete left bundle branch block
Others (eg, complete right bundle branch block and intraventricular conduction disturbance)
Abandoned HBP (RV pacing), yes/ no
Selective HBP/nonselective HBP
HBP threshold, V/0.5 ms or V/1.0 ms
RV pacing threshold, V/0.5 ms or V/1.0 ms
His injury current
Atrial/R wave sensing, mV/mV
Procedure time (total procedure time and time from His bundle mapping to final screwing), min
Pacing morphology after procedure (selective HBP/nonselective HBP/RV pacing)
Pre QRS duration, ms
Postpacing QRS duration. ms
In‐hospital complications (perforation, pneumothorax, lead revision, and reoperation)

Abbreviations: HBP, His bundle pacing; RV, right ventricular.

### 2.4 HBP implantation

The HBP procedure is performed in accordance with the standard method. After obtaining venous access, the specialized pacing lead (Select Secure 3830; Medtronic Inc.) and the sheath (C315His; Medtronic, Inc.) are advanced to the anterior or mid septum. When the electrograms from the His lead can identify the near‐field His electrogram, pacing from the lead is applied from 5.0 V at a 1 ms width. We check the 12‐lead surface electrograms simultaneously during pacing to facilitate His bundle capture or right ventricular capture. The pacing output is gradually decreased to the minimum output; thereafter, the pacing morphology is assessed. In general, a pacing threshold for His bundle capture (selective or nonselective HBP) of <2.0 V at a 1 milliseconds width is considered acceptable. Moreover, absence of far‐field atrial sensing and higher ventricular wave sensing are also important for the decision‐making regarding fixation of the His lead. Otherwise, the His lead position is changed, and the above mentioned His mapping was repeated until the acceptable criteria are achieved. However, in cases of a higher pacing threshold of the His bundle or noncapture of the His bundle after repetitive mapping and fixation, the pacing lead is fixed to the right ventricular septum, which is considered the traditional position with acceptable pacing and sensing parameters.

The pacing morphology was classified as selective HBP, nonselective HBP, and RVP in this study. The pacing morphology was defined according to the recent criteria proposed by a multicenter HBP collaborative working group.^18^ Selective HBP is defined as ventricular activation exclusively over the His‐Purkinje conduction system, with only capture of the tissue of the His bundle. The local ventricular electrogram in the HBP lead is recorded to differentiate from the pacing artifact. The duration from the stimulus to QRS onset is usually similar to the native His‐QRS onset interval; however, the interval from the stimulus to QRS onset may be shorter in case of an impaired His‐Purkinje conduction system and when capturing the distal segment of the conduction system. Additionally, paced QRS morphology was similar to the native QRS morphology, expect for patients with bundle branch block or escape rhythm. Nonselective HBP is defined as the capture of both His bundle and local ventricular myocardium at the pacing site and involves of two distinct pacing thresholds for the His bundle capture and RV capture. No isoelectric interval duration between the pacing stimulus and QRS onset is observed with the presence of a pseudo‐delta wave. Additional definitions of the HBP for the patients with His‐Purkinje conduction disease with or without bundle branch block correction are provided in the criteria.^18^

### 2.5 Follow‐up evaluation after implantation

During admission, data on the occurrence of complications related to pacemaker implantation are collected, if any. After discharge, the patients are followed up at a device clinic in each institution. At the 6 months follow‐up, the device parameters, including HBP threshold, pacing output, frequency of ventricular pacing, presence of an increase in the His bundle threshold, and pacing morphology (selective HBP/nonselective HBP/RVP), are evaluated (Table 2). Echocardiographic and electrocardiographic findings (eg, QRS duration), and laboratory data are also collected. Moreover, clinical events (eg, heart failure hospitalization, all‐cause death, and upgrade to CRT) and complications (eg, perforation, pneumothorax, lead revision, and reoperation) are assessed. We also evaluated the occurrence of an abandoned HBP and its cause during the follow‐up period. The patients are then scheduled to visit the device clinic every 6‐12 months in each institution.

**Table 2:** Parameters at the follow‐up evaluation after implantation

Parameters
6 months after implantation
Echocardiographic findings
Left ventricular ejection fraction, %
Left atrial diameter, mm
Left ventricular endo‐diastolic/‐systolic diameter, mm
Left ventricular endo‐diastolic/‐systolic volume, mL
Laboratory data
B‐type natriuretic peptide level, pg/dL
NT‐pro B‐type natriuretic peptide level, pg/dL
QRS duration on electrocardiogram, ms
Pacing morphology (selective HBP/nonselective HBP/RV pacing)
HBP threshold, V/0.5 ms or V/1.0 ms
RV pacing threshold, V/0.5 ms or V/1.0 ms
Increase in HBP threshold (+1V from baseline)
Pacing output, V/0.5 ms or V/1.0 ms
Frequency of ventricular pacing (from baseline), %
Complications (perforation, pneumothorax, lead revision, and reoperation)
Clinical events (heart failure hospitalization, all‐cause death, and upgrade to CRT)
Abandoned HBP (RV pacing), yes/no
3 years after implantation
Pacing morphology (selective HBP/nonselective HBP/RV pacing)
HBP threshold, V/0.5 ms or V/1.0 ms
RV pacing threshold, V/0.5 ms or V/1.0 ms
Complications (perforation, pneumothorax, lead revision, and reoperation)
Clinical events (heart failure hospitalization, all‐cause death, and upgrade to CRT)
Abandoned HBP (RV pacing), yes/no

Abbreviations: CRT, cardiac resynchronization therapy; HBP, His bundle pacing; RV, right ventricular

**Figure 1:**
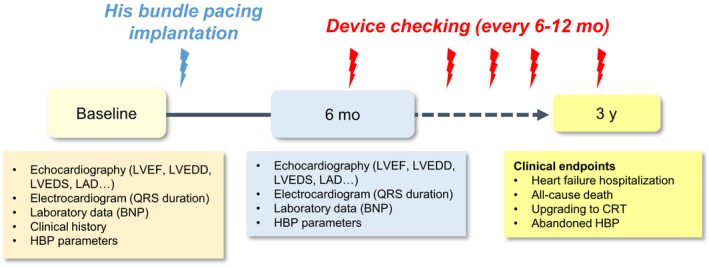
Entire study protocol and follow‐up schedule in the study. Abbreviations: BNP, B‐type natriuretic peptide; CRT, cardiac resynchronization therapy; HBP, His bundle pacing; LAD, left atrial diameter; LVEDD, left ventricular endo‐systolic diameter; LVEDS, left ventricular endo‐systolic diameter; LVEF, left ventricular ejection fraction

At 3 years after implantation, the pacing morphology, HBP threshold, and any occurrence of complications are assessed (Table 2). The clinical events of heart failure hospitalization, all‐cause death, upgrade to CRT, and HBP abandonment the follow‐up period are evaluated. The entire study protocol and follow‐up schedule are summarized in Figure 1.

### 2.6 Endpoints

The primary endpoints were all‐cause death, heart failure hospitalization, and upgrade to CRT after the 3‐year follow‐up (long‐term prognosis). The secondary endpoint was changes in cardiac function based on echocardiographic findings and laboratory data at 6 months after the procedure (short‐term prognosis).

## 3 RESULTS

The results are currently under investigation.

## 4 DISCUSSION

In Japan, HBP was introduced in 2017, and the number of HBP implantations performed for conduction diseases has been gradually increasing. There were several reports of unique observations in patients who received HBP, and studies with small sample sizes in single centers in Japan.^19^, ^20^, ^21^, ^22^, ^23^, ^24^ However, the long‐term performance, efficacy, and safety of HBP in Japanese patients are unknown.

Conversely, several recent reports demonstrated favorable outcomes with high success rates of HBP in the United States and Europe. The acute technical success rate of HBP was reported as approximately 90% in more than 100 experienced cases.^6^, ^14^, ^15^ However, one study that investigated 21 patients with heart failure and reduced ejection fraction who were suitable candidates for CRT reported a slightly low acute success rate of HBP (76%); this may indicate a difficulty in HBP application for complex patients with several comorbidities even in specialized cardiovascular centers.^9^ Another study showed an acute success rate of 75% for the first HBP implantation by electrophysiologists and lower achievement of HBP in patients with bundle branch block and complete heart block, indicating the need for some learning curve for HBP even among electrophysiologists.^25^ Moreover, to date, the maximum follow‐up period after HBP is 5 years, and most studies have used a follow‐up duration of several years.^13^ At the chronic phase after HBP, 5 of 100 patients (5%) required lead revision during a mean follow‐up period of 19 months.^15^ The causes were lead dislodgement, loss of capture, and increase in the pacing threshold. Another study reported that 14 of 304 patients (4.2%) who received HBP required lead revision during a mean follow‐up period of 754 days in an experienced institution.^6^ A recent study with long‐term follow‐up showed a significantly higher incidence of lead revision and generator replacement in the HBP group than in the RVP group.^13^ These reported rates of lead revision do not seem to be high, but are not low enough in reference to those associated with traditional RVP; this indicates the need for a careful and closed follow‐up after HBP implantation.

The recently updated 2018 JCS/JHRS guideline for nonpharmacotherapy of cardiac arrhythmias did not recommend or provide any indications of HBP officially owing to less accumulated evidence and on the outcomes, especially safety of HBP.^17^ This multi‐center observational study could provide helpful information on the outcomes and prognosis of the patients undergoing HBP in Japan in clinical settings.

## 5 CONCLUSIONS

We conducted this multicenter observational study with a long follow‐up period in patients undergoing HBP in Japan. The cumulative results, including the prognosis and changes in cardiac function, of the large number of patients would be helpful for establishing of further evidence on HBP in this area, and consequently allow accurate management and treatment of patients undergoing HBP.

## CONFLICT OF INTEREST

Satoshi Yanagisawa and Rei Shibata are affiliated with a department sponsored by Medtronic Japan. Other authors have no conflict of interest.
